# Discovery of a Monoclonal Antibody That Targets Cell-Surface Pseudaminic Acid of *Acinetobacter baumannii* with Direct Bactericidal Effect

**DOI:** 10.1021/acscentsci.3c01507

**Published:** 2024-02-07

**Authors:** Xuemei Yang, Ruohan Wei, Han Liu, Tongyao Wei, Ping Zeng, Yan Chu Cheung, Heng Heng, Edward Waichi Chan, Xuechen Li, Sheng Chen

**Affiliations:** † State Key Lab of Chemical Biology and Drug Discovery and the Department of Food Science and Nutrition, The Hong Kong Polytechnic University, Hung Hom 999077, Hong Kong SAR; ‡ Shenzhen Key lab for Food Biological Safety Control, The Hong Kong Polytechnic University Shenzhen Research Institute, Shenzhen 518000, China; § Department of Chemistry, the State Key Laboratory of Synthetic Chemistry, The University of Hong Kong, Pokfulam Road, Hong Kong 999077, Hong Kong SAR; ∥ School of Pharmacy, Faculty of Medicine, The Chinese University of Hong Kong, Shatin 999077, Hong Kong SAR; ⊥ Department of Infectious Diseases and Public Health, Jockey Club College of Veterinary Medicine and Life Sciences, City University of Hong Kong, Kowloon Tong 999077, Hong Kong SAR

## Abstract

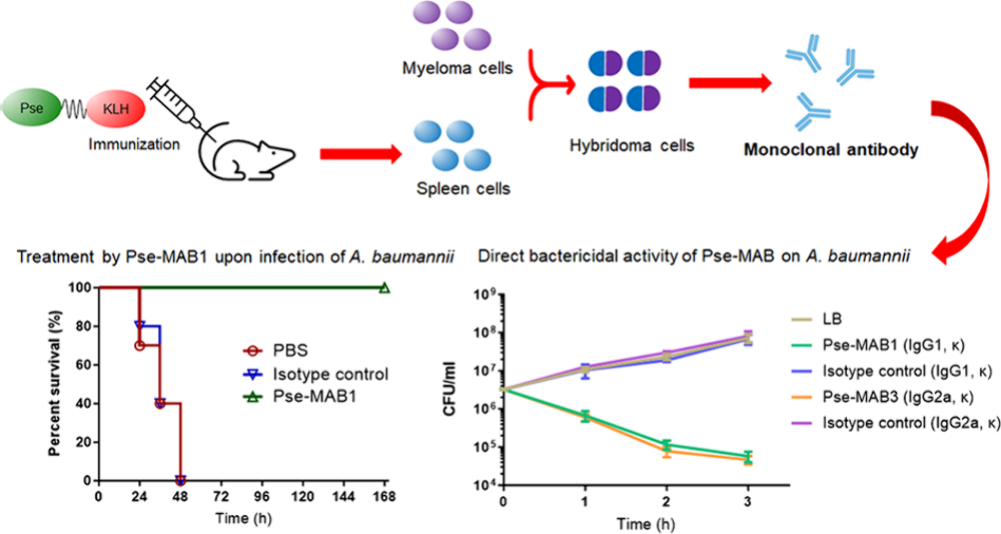

The therapeutic effects of antibodies include neutralization of pathogens, activation of the host complement system, and facilitation of phagocytosis of pathogens. However, antibody alone has never been shown to exhibit bactericidal activity. In this study, we developed a monoclonal antibody that targets the bacterial cell surface component Pseudaminic acid (Pse). This monoclonal antibody, Pse-MAB1, exhibited direct bactericidal activity on *Acinetobacter baumannii* strains, even in the absence of the host complements or other immune factors, and was able to confer a protective effect against *A. baumannii* infections in mice. This study provides new insight into the potential of developing monoclonal antibody-based antimicrobial therapy of multidrug resistant bacterial infections, especially those which occurred among immunocompromised patients.

## Abstract 2

Monoclonal antibody targeting Pse was generated by immunization of mice with synthesized Pse-KLH conjugates. Pse-MAB1 could mediate direct bacterial cell death and promote *in vivo* protection against *A. baumannii* infection in mice.

## Introduction

*Acinetobacter baumannii* is an opportunistic pathogen which can cause both hospital- and community-acquired infections of high mortality, including pneumonia, bloodstream infections, meningitis, urinary tract infections, and wound and surgical site infections.^1^ Multidrug resistant (MDR) *A. baumannii* strains have disseminated worldwide and possess exceptional ability to further acquire exogenous resistance-encoding genetic elements.^2−4^ The increasing incidence of resistance of such strains to the last sort of antibiotics carbapenem, colistin, and tigecycline results in lack of treatment options and poor clinical outcome.^5^ Hence, novel antimicrobial approach is urgently required for treatment of MDR *A. baumannii* infections. Immunotherapies that utilize monoclonal antibodies have exhibited wide potential in the anticancer, autoimmune, and antiviral fields, yet a therapeutic antibody for treatment of bacterial infection is not common.^6^ Currently known antibacterial antibodies exert their effects mainly through complement-mediated lysis, phagocytes opsonization, and cell-mediated immunity.^6^ Bactericidal antibodies whose action does not involve complements or other immune factors have not been reported. A recent study reported a monoclonal antibody that exhibits direct bactericidal effect on the Δ*waaD Escherichiacoli* mutant strain with truncated lipopolysaccharides (LPS).^7,8^ This monoclonal antibody directly binds to BamA, the β-barrel assembly machine of *E. coli*, and inhibits its β-barrel folding activity, thus inducing periplasmic stress and disrupting outer membrane integrity. Although this monoclonal antibody did not cause bacterial cell death, this finding suggests that monoclonal antibody could exhibit direct bactericidal effect when targeting specific proteins or cellular components.

Pseudaminic acid (Pse) is a nine-membered ketoaldonic acid derivative that belongs to the nonulosonic acid family. Pse was first discovered as a component of lipopolysaccharides (LPS) in *Pseudonomas aeruginosa* and *Shigella boydii* in 1984.^9^ Since then, Pse has also been identified in *Vibrio vulnificus* as a component of LPS^10^ and in *Acinetobacter baumannii* as a component of the capsular polysaccharide (CPS).^11^ Except for being a component of cell surface-associated glycans, Pse has also been found to play a role in modifying the flagella of the Gram-negative bacteria *Aeromonas caviae*,^12^*Helicobacter pylori*,^13^ and *Campylobacter jejuni*.^14^ More recently, Pse has also been discovered in the Gram-positive bacterium *Bacillus thuringiensis*.^15^ Furthermore, post-translational Pse modification of flagellin has been found to be necessary for flagella biogenesis in *C. jejuni*, which plays an important role in bacterial mobility and colonization.^14^ Therefore, Pse has emerged as an attractive target for the development of new antimicrobial therapeutics in recent years.^16^ In our previous studies, we have developed an approach to synthesize bacterial Pse and its derivates^17,18^ and found that Pse is widely distributed in various bacterial species such as *P. aeruginosa*, *V. vulnificus*, and *A. baumanii*.^19^ Furthermore, the artificially synthesized Pse was conjugated to the carrier protein CRM197 for development of a vaccine that could stimulate expression of strong immune response in mice for protection against infections caused by Pse-producing *A. baumannii*.^20^ This previous work prompted us to further develop the monoclonal antibody (mAb) against Pse and use such antibody in a novel approach to treat infections caused by various bacterial pathogens, in particular, MDR *A. baumannii*, which often cause untreatable infections due to the lack of effective antibiotics. In this study, we developed a new strategy to produce mAbs that target Pse. Surprisingly, we found that this antibody exhibited strong killing effect on *A. baumannii*, including the MDR strains in the absence of complement or other immune factors. This is the first report of a monoclonal antibacterial antibody that exhibits direct bactericidal effect against clinical bacterial strains. The findings of this work provide important insight into the development of novel antibody-based therapies to treat bacterial infections regardless of the drug susceptibility status of the causative agents.

## Results

### Synthesis of Pse-KLH and Pse-BSA Conjugates

**Figure 1:**
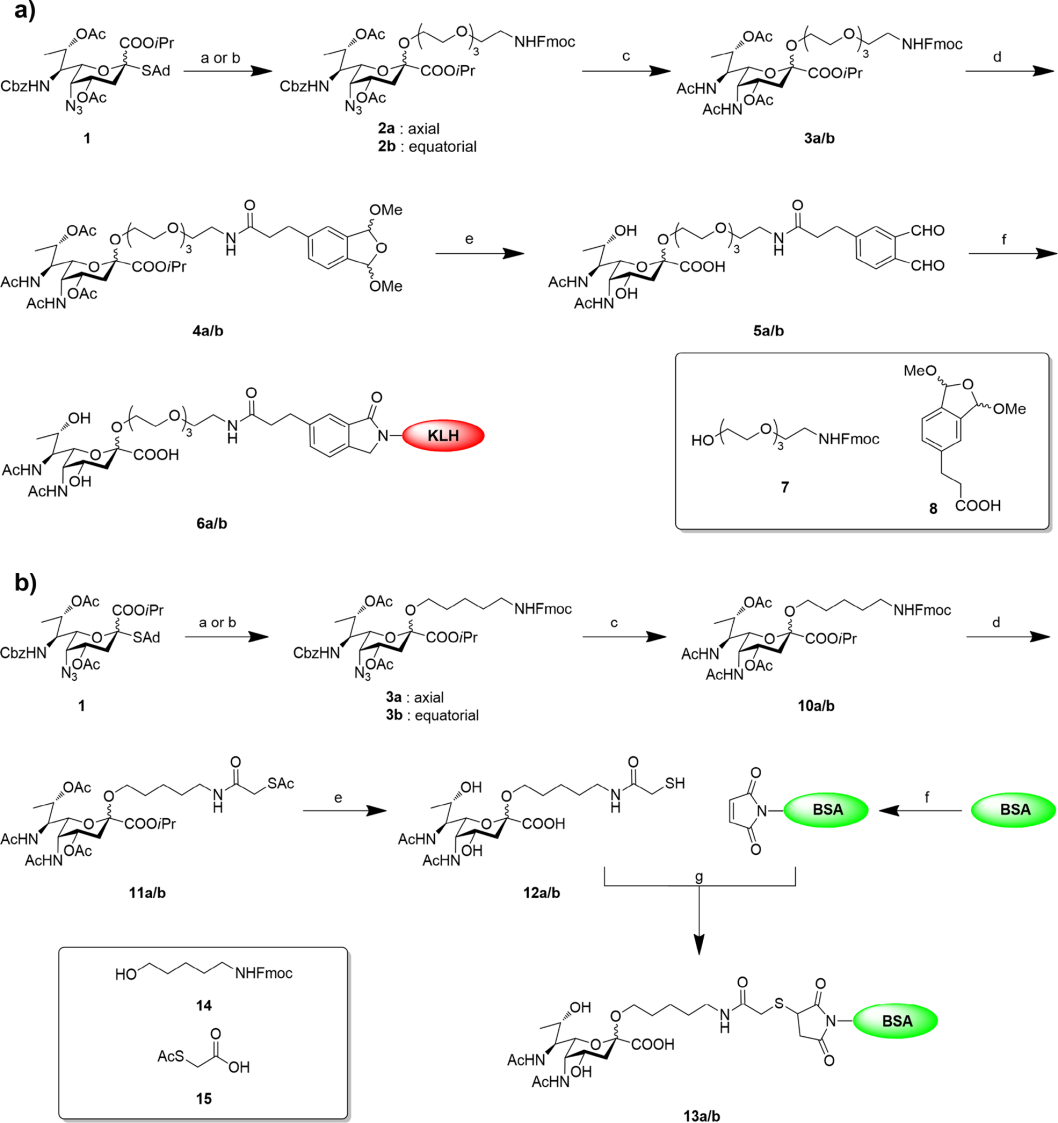
**Design and synthesis of Pse-KLH and Pse-BSA conjugates.** (a) Synthesis of the Pse-KLH conjugate. Reagents and conditions: (a) NIS, TfOH, DMF, DCM, acceptor **7**, −40 °C, 6 h, 80%. (b) NIS, TfOH, DCM, MeCN, −78 °C, 6 h, 77%. (c) Pd/C, NH_4_OAc, DCM-MeOH, H_2_ (1 atm), 30 min, then NMM, Ac_2_O, 1 h, 77% for **3a**, 73% for **3b**. (d) (i) DEA-MeCN, 30 min; (ii) acid **8**, EDCI, DIEA, DCM, 87% for **4a**, 85% for **4b**. (e) (i) LiOH, MeOH-THF-H_2_O, 24 h; (ii) 10%HOAc (aq), 2 h, 75% for **6a/b** over 2 steps. (f) KLH carrier protein, PBS buffer (pH 7.4), rt, 2 h. (b) Synthesis of Pse-BSA conjugate. Reagents and conditions: (a) NIS, TfOH, DMF, DCM, acceptor **14**, −40 °C, 6 h, 82%. (b) NIS, TfOH, DCM, MeCN, −78 °C, 6 h, 78%. (c) Pd/C, NH_4_OAc, DCM-MeOH, H_2_ (1 atm), 30 min, then NMM, Ac_2_O, 1 h, 78% for **10a**, 77% for **10b**. (d) (i) DEA-MeCN, 30 min; (ii) acid **15**, EDCI, DIEA, DCM, 87% for **11a**, 85% for **11b**. (e) LiOH, MeOH-THF-H_2_O, 24 h, 79% for **12a**, 83% for **12b**. (f) Sulfo-EMCS, PBS (pH 8.0), rt, 1.5 h. (g) PBS (pH 7.4), rt, 2 h.

Based on our previous research in the *de novo* synthesis of pseudaminic acid derivatives and highly stereoselective pseudaminylation,^17,18^ we synthesized poly(ethylene glycol) (PEG)-modified pseudaminic acid **2a/b** from glycosyl donor **1** and Fmoc-protected PEG linker **7** in both α (axial) and β (equatorial) forms with good yield (Figure 1). The azido and Cbz group could be selectively removed without affecting the Fmoc group by hydrogenolysis in the presence of ammonium acetate; the liberated amines were then acetylated by acetic anhydride to give **4a/b** (Figure 1a). *ortho*-Phthalaldehyde (OPA) was reported to be highly reactive and selective toward primary amines and could be used in the conjugation to carrier proteins.^20^ Considering the acid lability of the pseudaminyl linkage and the ability of OPA to form polymers, we coupled compound **8** to the amine after Fmoc removal under the mild conditions which facilitate freeing the phthaldehyde. Finally, deprotection was conducted by treating **4a/b** with lithium hydroxide, followed by 10% aqueous acetic acid solution to give the Pse-OPA linker **5a/b**. The resultant linker subsequently reacted with KLH carrier protein in PBS (pH 7.4) to generate Pse-KLH conjugate **6a/b**.

In order to confirm that the generated antibody specially targets Pse, we also synthesized the BSA-Pse conjugate (Figure 1b). The connection strategy was changed to an alkyl linker and the thiol-maleimide method to minimize unexpected recognition of the carrier protein and linker attached to the antibody. Compound **10a/b** was obtained using the same strategy as described above, and 2-(acetylthio)acetic acid was coupled after Fmoc removal to give **11a/b**. Finally, the Pse-thiol linker **12a/b** was obtained by basic hydrolysis of esters. Commercially available BSA was treated with *N*-(ε-maleimidocaproxy)sulfosuccimide ester (sulfo-EMCS) in PBS (pH 8.0) to install a maleimide onto the protein, which further reacted with the Pse-thiol linker in PBS (pH 7.4) to give BSA-Pse conjugate **13a/b**.

### Development of Mouse Monoclonal Antibodies Target Pse

**Figure 2:**
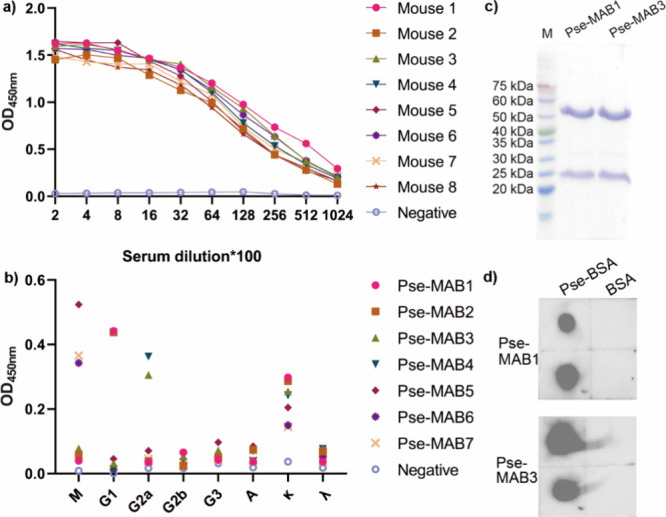
**Development of mouse monoclonal antibodies targeting Pse.** (a) Immune response of BALB/c mice that received immunization of the Pse-KLH conjugate. Sera collected on day 55 were 2-fold diluted from 200 and subjected to determination of the end point titer of Pse-specific antibodies by ELISA. (b) Isotyping of Pse-specific antibodies in cell supernatants of the 7 hybridoma cell lines by ELISA. HRP-conjugated goat antimouse IgM, IgG1, IgG2a, IgG2b, IgG3, IgA, κ, and λ were used to type the Pse-specific antibodies in the cell supernatant. (c) SDS-PAGE of purified Pse-MAB1 and Pse-MAB3. M: marker. (d) Dot-blot of Pse-MAB1 and Pse-MAB3 against Pse-BSA conjugate. Pse-BSA conjugate and BSA as control were dotted onto the membrane, incubated with Pse-MAB1 or Pse-MAB3 as primary antibodies, and HRP conjugated goat antimouse IgG as secondary antibody.

To develop monoclonal antibodies that target Pse, eight female BALB/c mice were immunized with the Pse-KLH conjugate formulated with the Freund’s complete adjuvant via subcutaneous injection. The control group received KLH mixed with the Freund’s complete adjuvant in PBS. On days 14, 28, and 49, the mice received a booster injection. Blood was collected on day 55 to retrieve serum, and Pse-specific antibody titers in sera were tested by ELISA using Pse-BSA conjugate as captures. All mice produced strong antibody responses to Pse-BSA (Figure 2a). The best-responding mouse (Mouse 1) was boosted two more times, on days 98 and 110, respectively, to develop monoclonal antibodies. Its spleen was collected, and spleen cells were then harvested for hybridoma fusion with the SP2/0 cells. Finally, a total of 930 monoclonal cells were picked up from the HAT selective medium and cultured in 96-well plates. The Pse-specific antibodies in the cell supernatants were tested by ELISA using Pse-BSA conjugates as captures. After two rounds of screening, a total of 7 hybridoma cell lines producing Pse-specific antibodies were established (Figure S1). Isotyping of these 7 Pse-specific monoclonal antibodies (Pse-MABs) in the cell supernatants was performed by ELISA. Pse-MAB1 and Pse-MAB2 were identified as IgG1, Pse-MAB3 and Pse-MAB4 were identified as IgG2a, while Pse-MAB5, Pse-MAB6, and Pse-MAB7 were identified as IgM (Figure 2b). All of these 7 antibodies were identified as κ type (Figure 2b). We then purified a large amount of monoclonal antibodies for further characterization. Hybridoma cell lines 1 and 3 were intraperitoneally injected into female BALB/c mice, successively. Ascites were collected, and the IgG mAbs were further purified using Protein G agarose, HiTrap Q HP column, and Superdex 75 size column by steps to obtain pure mAbs (Figure 2c). The purified mAbs were changed to buffer 20 mM Tris-HCl, pH 7.4, 40 mM NaCl and stored at −80 °C. The epitope of these two mAbs was further confirmed by dot-blot. Both Pse-MAB1 and Pse-MAB3 were found to be able to bind Pse-BSA conjugate dotted on the membrane (Figure 2d).

### Direct Bactericidal Effects of Pse-MABs to *A. baumannii*

**Figure 3:**
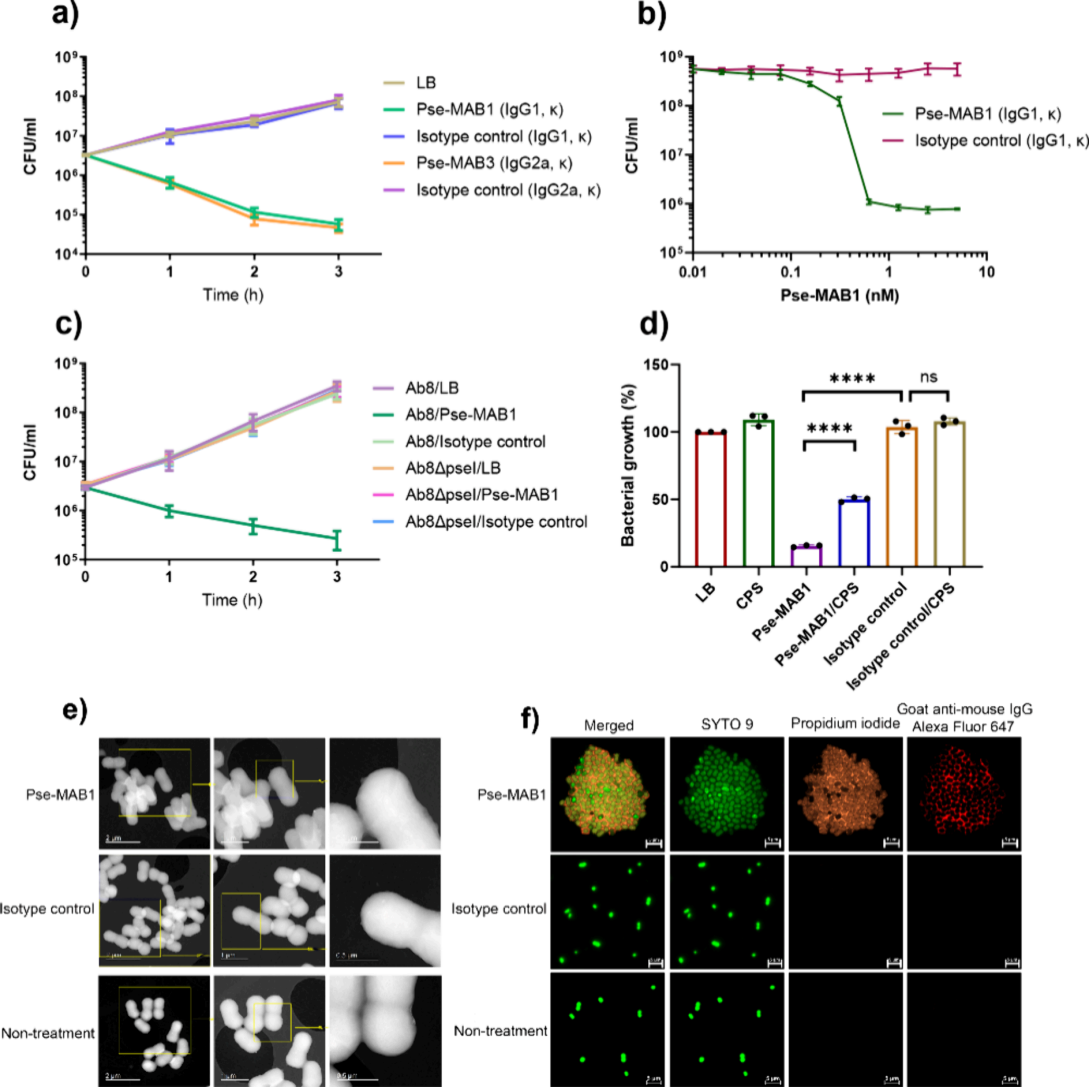
**Growth inhibition of*****A. baumannii*****strain caused by Pse-MAB.** (a) Growth curve of *A. baumannii* strain Ab-00.191 in the presence of Pse-MAB1, isotype control (mouse IgG1, κ), Pse-MAB3, or isotype control (mouse IgG2a, κ) (1 nM). CFUs were measured at 0, 1, 2, 3 h after the addition of Pse-MAB1 and isotype control. (b) Growth inhibition of *A. baumannii* strain Ab-00.191 in the presence of Pse-MAB1 or isotype control (mouse IgG1, κ). CFUs were measured at 3 h intervals after addition of different concentrations of Pse-MAB1 and isotype control. (c) Growth curve of *A. baumannii* strain Ab8 and the *pseI* gene deletion mutant Ab8*ΔpseI* in the presence of Pse-MAB1 or isotype control (mouse IgG1, κ) (1 nM). (d) Growth of *A. baumannii* strain Ab-00.191 in the presence of Pse-MAB1 (1 nM) or isotype control (mouse IgG1, κ) treated with CPS. Statistical analysis was performed with the unpaired two-sided Student’s *t*-test using GraphPad Prism (San Diego, CA). *P* < 0.0001 (****). (e) STEM of *A. baumannii* strain Ab-00.191 treated with Pse-MAB1 or isotype control for 3 h. (f) Fluorescence microscopy of *A. baumannii* strain Ab-00.191 exposed to Pse-MAB1 or isotype control for 0.5 h. A representative image is shown in (e, f).

We then determined the antibacterial effects of the purified Pse-specific monoclonal antibodies to Pse-producing bacterial strains. Incubation of purified Pse-MAB1 (IgG1, κ) with Pse-producing *A. baumannii* strain Ab-00.191 caused a time-dependent decrease in the number of viable bacterial cells. At 3 h after addition of Pse-MAB1, the number of colony-forming units (CFUs) decreased 3 logs when compared to the strains incubated with the isotype control antibody (mouse IgG1, κ), demonstrating that Pse-MAB1 exerted bactericidal effect on this strain (Figure 3a). Furthermore, growth inhibition caused by Pse-MAB1 was found to be concentration-dependent, with ∼0.6 nM mAb being required to achieve complete inhibition of bacterial growth (Figure 3b). Pse-MAB1 exhibited no killing effects on the non-Pse-producing *A. baumannii* strain Ab-11.854, indicating that Pse might be the target of Pse-MAB1 through which the bactericidal effect of the antibody is mediated (Figure S2). We then tested Pse-MAB1 in another Pse-producing *A. baumannii* strain, namely, Ab1, and a similar bactericidal effect was observed (Figure S2). Interestingly, although Pse-MAB1 was shown to react with the Pse-producing *V. vulnificus* strain Vv3 and *P. aeruginosa* strain PA12, bactericidal effect was not observed upon incubation of these strains with Pse-MAB1, suggesting that the bactericidal effect of this monoclonal antibody was highly specific to *A. baumannii* (Figure S2). The quantity of Pse production and the components of Pse being targeted (such as CPS for the *A. baumannii* strain, LPS for *V. vulnificus* and *P. aeruginosa* strains) might affect the bactericidal effect. We also tested another type of the Pse monoclonal antibody, Pse-MAB3 (IgG2a), which also exhibited similar bactericidal effects to *A. baumannii* strain Ab-00.191 (Figure 3a). To confirm that the Pse molecule was the target of Pse-MAB1, we generated a *pseI* gene deletion mutant in the susceptible strain *A. baumannii* Ab8 which also produced Pse. It was found that Pse-MAB1 exhibited bactericidal effects against the wild-type strain Ab8 but not the *pseI* gene deletion mutant Ab8ΔpseI (Figure 3c), indicating that the Pse molecule was the target of Pse-MAB1. As Pse exists as a component of the repeat unit of the capsule (CPS) of *A. baumannii*, we further extracted the CPS from strain Ab-00.191 and incubated with Pse-MAB1 prior to the killing assay. The results showed that 84.8% of the bacteria were dead when treated with Pse-MAB1, whereas only 50.8% was dead when treated with CPS-incubated Pse-MAB1 (Figure 3d). These data suggested that the monoclonal antibody Pse-MAB1 targeted the surface Pse in the CPS of *A. baumannii* strains and mediated bacterial cell death.

Aggregation was observed to occur when *A. baumannii* strain Ab-00.191 was incubated with Pse-MAB1 during culture in LB broth, while there was no aggregation of *A. baumannii* strain Ab-00.191 with the isotype control. Scanning transmission electron microscopy (STEM) was then performed on these bacterial cells, with results showing that aggregation of *A. baumannii* strain Ab-00.191 occurred after addition of Pse-MAB1 but not the isotype control (Figure 3e). We therefore concluded that the Pse epitope of the Pse-MAB1 interacted with the surface Pse of *A. baumannii* strain and mediated bacterial aggregation. Next, a live and dead staining experiment was performed upon treatment of *A. baumannii* strain Ab-00.191 cells with Pse-MAB1. The dye SYTO 9 stained both live and dead bacterial cells and propidium iodide (PI) stained dead bacterial cells only and caused a reduction in the fluorescence level of the SYTO 9 stain when both dyes were present.^21^ Therefore, we were able to distinguish between the dead and live bacterial cells by using these two dyes. Furthermore, Pse-MAB1 was incubated with Goat Anti-Mouse IgG H&L (Alexa Fluor 647) in advance so that it could be observed using fluorescence imaging. As shown in Figure 3f, the *A. baumannii* strain Ab-00.191 cells were found to aggregate when treated with Pse-MAB1, and more than 99% of the cells were found to be dead, which was consistent with the observations described above. The Pse-MAB1 was found to be located between the bacterial cells (Figure 3f). A video was generated by capturing continuous images for a duration of 10 min and interval of 10 s (Supporting Information File 2). The cells were found to cluster immediately after addition of Pse-MAB1. When treated with the isotype control, the *A. baumannii* strain Ab-00.191 cells remained dispersed, and most were found to be alive in a manner similar to that of the culture grown in LB broth (Figure 3f). Agglutination is a common phenomenon observable when targeted bacterial cells come into contact with specific monoclonal antibodies which do not cause bacterial death.^22^ Nevertheless, our findings support the view that monoclonal antibody exhibits some degree of bacteriocidic effect, which varies between different antibody–bacteria combinations.

### *In Vivo* Therapeutic Effects of Pse-MAB1 against *A. baumannii* Infections

**Figure 4:**
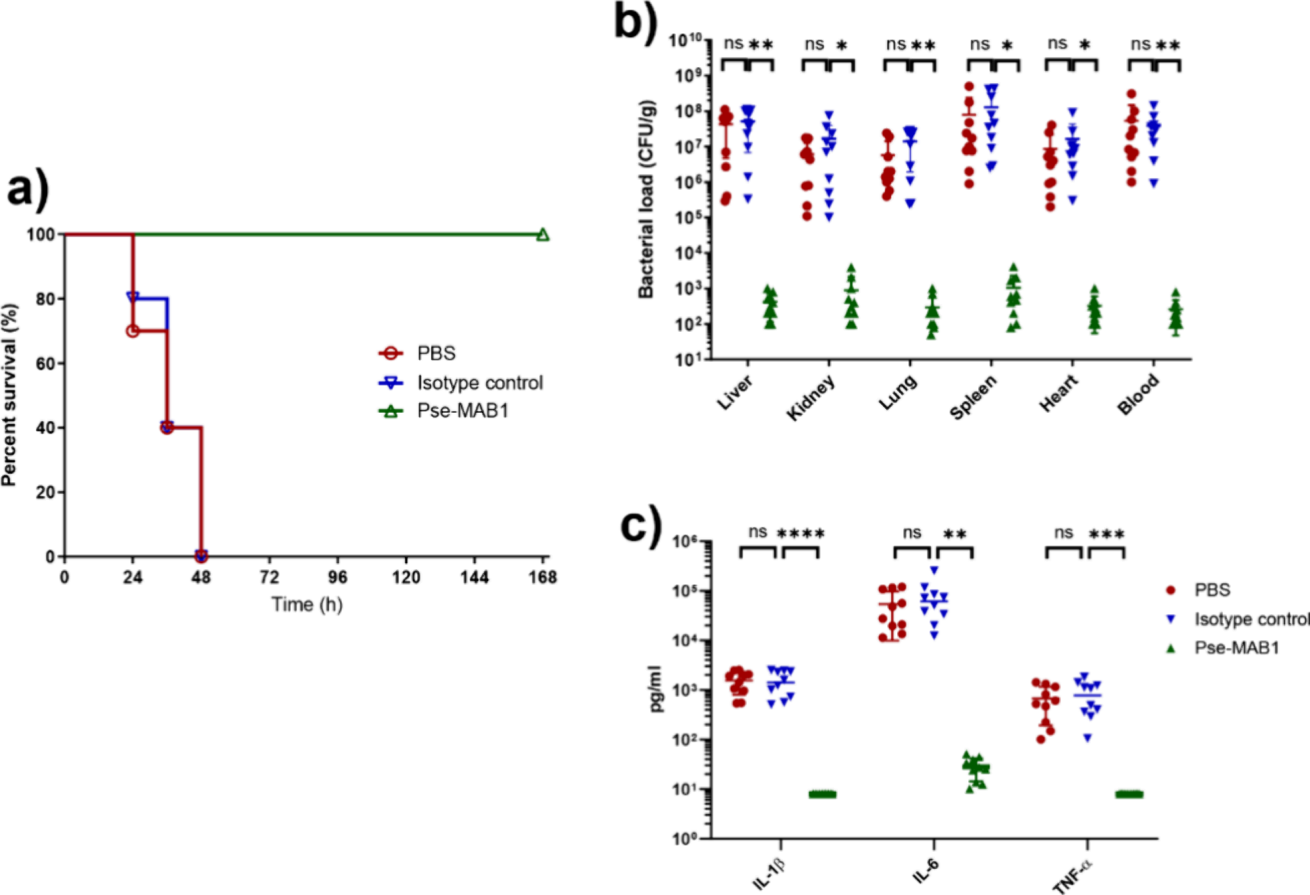
**Pse-MAB1 confers*****in vivo*****protection in a mouse infection model.** (a) Survival of mice (*n* = 10) infected by 4 × 10^7^ CFU of *A. baumannii* strain Ab-00.191 receiving Pse-MAB1 treatment or isotype control at 168 h. (b) Bacterial loads in liver, kidney, lung, spleen, heart, and blood collected from mice at 24 h post infection by 4 × 10^7^ CFU of *A. baumannii* strain Ab-00.191 receiving Pse-MAB1 treatment or isotype control (*n* = 10 mice/group). (c) Serum levels of proinflammatory cytokines IL-1β, IL-6, and TNF-α of mice at 24 h post infection by 4 × 10^7^ CFU of *A. baumannii* strain Ab-00.191 receiving Pse-MAB1 treatment or isotype control (*n* = 10 mice/group). Statistical analysis was performed with the unpaired two-sided Student’s *t*-test using GraphPad Prism (San Diego, CA). *P* < 0.05 (*), *P* < 0.01 (**), *P* < 0.001 (***), *P* < 0.0001 (****).

The *in vivo* protective effects by which Pse-MAB1 confers against *A. baumannii* infections was determined in a mouse infection model. Ten mice in a group were inoculated with 4.0 × 10^7^ CFU (2 × LD50) of *A. baumannii* strain Ab-00.191, followed by injection with PBS, the isotype control (IgG1, κ), and Pse-MAB1 as treatment, respectively. The survival rate of mice was 0% when treated with PBS as well as the isotype control, whereas a rate of 100% was recorded when treated with Pse-MAB1 (*P* < 0.0001) (Figure 4a), indicating that Pse-MAB1 conferred *in vivo* protective effect against *A. baumannii* infection. Furthermore, tissue bacterial loads in mice infected with *A. baumannii* strain Ab-00.191 and then treated with PBS, isotype control, and Pse-MAB1 were determined, and the results showed a significant decrease in the group treated with Pse-MAB1, when compared to the isotype control (Figure 4b). Meanwhile, serum levels of proinflammatory cytokines IL-1β, IL-6, and TNF-α were measured. Consistent with the protective efficacy, the levels of all three cytokines were significantly decreased when treated with Pse-MAB1 (Figure 4c). Apart from the direct bactericidal effects, Pse-MAB1 that bind to the bacteria *in vivo* might also activate the host immune and complement system, thus assisting in bacterial killing.

## Discussion

Immunotherapies via the use of monoclonal antibodies have a wide range of applications in treatment of various diseases. These antibodies exert the biological activity by blocking protein-receptor interactions, while antibacterial antibodies exhibit their effects mainly through complement-mediated lysis, phagocytes opsonization, and cell-mediated immunity. A bactericidal antibody has rarely been reported. An example is an MAB targeting the β-barrel assembly machine of *E. coli*.^7^ This MAB directly binds to BamA and inhibits its β-barrel folding activity, inducing periplasmic stress and disrupting outer membrane integrity. However, this antibody could not directly bind to BamA protein due to the blockage by bacterial LPS. Therefore, its direct killing effect could only be seen in LPS knockout *E. coli* but not clinical *E. coli* strains. In this study, we synthesized Pse-KLH conjugates and developed Pse-specific MABs raised from mice. A surprising finding is that the monoclonal antibody Pse-MAB1 could mediate bacterial cell death in Pse-producing *A. baumannii* strains without any activating complements and could promote *in vivo* protection against *A. baumannii* infection in mice.

Our studies indicated that Pse-MAB1 targeted the Pse molecules on the surface CPS of *A. baumannii* strains, mediated bacterial aggregation, and triggered metabolic disorders and oxidative injury (data not shown), thereby causing bacterial cell death. It is then speculated that the aggregated bacterial cells then entered oxidative stress conditions, which resulted in overproduction of ROS. However, the metabolic dysregulation as a direct downstream function of Pse-MAB1 remained uncertain, as the interaction between Pse-MAB1 and the bacterial cell was immediate, and the metabolic change may be the result of dying cells. C. P. Ewing et al. has identified that the flagellin FlaA of *C. jejuni* is Pse-modified and the glycosylation is required for motile and autoagglutination.^23^*A. baumannii* lacks flagella but exhibits twitching or swarming motility by pili and exopolysaccharide.^24^ Another hypothesis is that Pse-MAB1 may bind to certain Pse-modified proteins, interfere with their functions, and mediate bacterial death. However, it is unable to verify at this stage, as the proteins remained uncharacterized yet. This is to be studied to better understand the biological functions of Pse in *A. baumannii*.

The monoclonal antibody Pse-MAB1 improved survival rate of mice upon infection by MDR *A. baumannii* and reduced bacterial loads and inflammatory responses. The protective ability of Pse-MAB1 makes it an effective treatment for *A. baumannii* infections. The biosynthesis loci of Pse have been identified in several clinically prevalent isotypes of *A. baumannii*, including KL2, KL6, KL16, KL23, KL31, KL33, KL42, KL46, KL58, KL77, KL81, KL90, KL93, and KL120.^25^ Moreover, KL2 is the most common serotype of *A. baumannii* and is highly associated with carbapenem resistance and virulence.^26^ Thus, the development of Pse-based monoclonal antibody therapy is attractive, especially for those with compromised immune systems. In conclusion, the discovery of the novel function of monoclonal antibodies extends its therapeutic applications to immunocompromised patients and opens new opportunities to study hitherto unknown bactericidal pathways.

## Material and Methods

Provided in Supporting Information.

## Supporting Information Available

Method details and Figures S1 & S2 (PDF)
A video was generated by capturing continuous images for a duration of 10 min and interval of 10 s (MP4)
