# Supplementary material for: Discovery of a Monoclonal Antibody That Targets Cell-Surface Pseudaminic Acid of Acinetobacter baumannii with Direct Bactericidal Effect
Source: ACS Cent Sci. 2024 Feb 7;10(2):439–46. doi: 10.1021/acscentsci.3c01507 (PMC10906240; doi:10.1021/acscentsci.3c01507)
Supplement: Supplementary file 1 — oc3c01507_si_001.pdf [file oc3c01507_si_001.pdf]

Supporting Information

Discovery of a monoclonal antibody that targets cell-surface pseudaminic acid of *Acinetobacter baumannii* with direct bactericidal effect

Xuemei Yang[1,2], Ruohan Wei,[3] Han Liu,[3] Tongyao Wei,[3] Ping Zeng[4], Yan Chu Cheung[1,5], Heng Heng[1,5], Edward Wai Chi Chan[2], Xuechen Li[3]* and Sheng Chen,[1,2]*

[1]State Key Lab of Chemical Biology and Drug Discovery and the Department of Food Science and Nutrition, The Hong Kong Polytechnic University, Hung Hom, Hong Kong SAR;

[2]Shenzhen Key lab for Food Biological Safety Control, The Hong Kong Polytechnic University Shenzhen Research Institute, Shenzhen, China;

[3]Department of Chemistry, the State Key Laboratory of Synthetic Chemistry, The University of Hong Kong, Pokfulam Road, Hong Kong SAR;

[4]School of Pharmacy, Faculty of Medicine, The Chinese University of Hong Kong, Shatin, Hong Kong SAR;

[5]Department of Infectious Diseases and Public Health, Jockey Club College of Veterinary Medicine and Life Sciences, City University of Hong Kong, Kowloon Tong, Hong Kong SAR;

Corresponding author: Sheng Chen, The Hong Kong Polytechnic University, Hong Kong, E-mail: sheng.chen@polyu.edu.hk; Xuechen Li, The University of Hong Kong, Hong Kong, E-mail: xuechenl@hku.hk

Supplementary Methods--S2

Supplementary Results---S8

References---S10

Supplementary Methods

Bacterial strains and mice used in animal experiments

Pse-producing *A. baumannii* strain Ab-00.191 and non-Pse-producing *A. baumannii* strain Ab-11.854 were reported in our previous studies [1, 2]. The strains were isolated from clinical samples, identified by the Vitek 2 system (bioMérieux, France) and confirmed by the matrix-assisted laser desorption/ionization-time of flight mass spectrometry apparatus (MALDI-TOF MS) (Bruker, Germany). The strains were further characterized using their genomic sequences. The genomic sequences have been updated to GenBank under BioProject No. PRJNA481426. Pse-producing *V. vulnificus* strain Vv3 and *P. aeruginosa* strain PA12 were reported in our previous study [1]. Strains used in this study were cultured in Luria-Bertani (LB) broth or Tryptic soy broth (TSB) at 37°C without additional instructions.

Female BALB/c inbred strains of mice (six to eight weeks old, ~20 g) were obtained from the Laboratory Animal Research Unit (LARU), City University of Hong Kong. Animals were handled in strict accordance with the Animals (Control of Experiments) Ordinance (Cap. 340), Hong Kong. All animal experiments were approved by the Animal Research Ethics Sub-Committee (ARESC) of City University of Hong Kong. Animals were housed under specific pathogen-free conditions during experiment. All efforts were made to minimize animal suffering.

Development of monoclonal antibodies (mAbs) targeting Pse

Female 6-8 weeks old inbred BALB/c mice were immunized with the Pse-KLH conjugates formulated with the Freund's complete adjuvant via subcutaneous injection. On days 14, 28 and 49, the mice received a booster injection. Blood was collected on day 55 to retrieve serum, and Pse-specific antibody titers in sera were tested using ELISA. The best-responding mouse

was boosted two more times on days 98 and 110. Its spleen cells were then harvested for hybridoma fusion with SP2/0 cells. A total of 93*10 monoclonal cells were picked up from the HAT selective medium and cultured in 96-well plates. An ELISA assay was performed to detect Pse-specific antibodies in the supernatants. After two rounds of screening, a total of 7 hybridoma cell lines producing Pse-specific antibodies were established.

ELISA assays to detect Pse-specific antibodies

Pse-specific antibodies in sera or cell supernatants were determined by ELISA as previously described [2]. Briefly, high binding 96 well polystyrene microtiter plates were coated with BSA-Pse, blocked with 2% BSA, and incubated with post-immune sera or cell supernatants. The plates were further incubated with horseradish peroxidase (HRP) conjugated goat anti-mouse antibody (Abcam, UK) and developed using tetramethylbenzidine (TMB). The reaction was stopped by adding 2% sulfuric acid and the absorbance was recorded at 450 nm. Pse-specific antibodies isotyping of IgA, IgG1, IgG2a, IgG2b, IgG3, IgM, κ or λ was performed using the SBA Clonotyping System-HRP for mouse (Southernbiotech, United States).

Purification of Pse-MAB1 and Pse-MAB3

Hybridoma cell lines 1 and 3 were intraperitoneally injected into female BALB/c mice, respectively. After one week, ascites from the mice were collected and centrifuged to remove tissues and cells. The IgG proteins from ascites were first purified using the protein G agarose pre-equilibrated with sodium phosphate buffer (20 mM, pH7.0), eluted with the glycine-HCl buffer (100 mM, pH2.7) and neutralized with the Tris-HCl buffer (1M, pH9.0). The IgG proteins were further purified using the HiTrap Q HP column pre-equilibrated with sodium phosphate buffer (20 mM, pH7.0) and gradient eluted with elution buffer (20 mM sodium phosphate, 500 mM NaCl, pH7.0). The collections were further loaded on the Superdex 75 size

column to obtain pure mAbs. The purity of mAbs was tested by SDS-PAGE. The purified mAbs were changed to buffer 20 mM Tris-HCl, pH 7.4, 40 mM NaCl and stored at -80 °C.

Dot-blot to detect Pse-specific antibodies

A volume of 2 μl Pse-BSA conjugate or BSA were doted on the PVDF membrane and blocked using 5% milk in TBST. The membrane was then incubated with Pse-MAB1 or Pse-MAB3 in 5% milk in TBST followed by 5 times washes with TBST. The membrane was further incubated with HRP conjugated goat anti-mouse antibody followed by 8 times washes with TBST. Finally, the membrane was incubated with a chemiluminescent substrate and imaged.

Antibody-mediated bactericidal activity assay

Bacterial cells were grown to exponential phase at 37 °C and diluted to OD600 of 0.01 with fresh LB broth. Upon addition of antibody Pse-MAB1, bacterial culture was incubated at 37 °C with shaking. At indicated timepoints, bacterial culture was serially diluted with saline and bacterial concentrations were determined by plating dilutions on LB agar plates. Each experiment was performed with three technical and three biological replicates. To determine the inhibition effect of capsule, Pse-MAB1 was pre-incubated with capsule before addition into the bacterial culture.

Scanning transmission electron microscopy (STEM)

Bacterial cells were grown to exponential phase at 37 °C and diluted to OD600 of 0.01 with fresh LB broth. Upon addition of antibody Pse-MAB1, bacterial culture was incubated at 37 °C for 3 hours with shaking. Bacterial cells were collected, washed with PBS and treated with 4% PMF overnight at 4 °C. Imaging was performed on Field Emission Electron Microscope STEM (JEOL Model JEM-2100F) (JEOL Ltd., Japan).

Microscopy

Pse-MAB1 was incubated with Goat Anti-Mouse IgG H&L (Alexa Fluor® 647) (Abcam, UK) at room temperature in advance to locate the antibody. 1 ml of exponential phase bacterial cells was collected, washed with PBS, incubated with treated Pse-MAB1 together with LIVE/DEAD BacLight Bacterial Viability Kit (Invitrogen, USA) at room temperature for 30 min. The staining kit was comprised of two components, SYTO 9 for staining all bacterial cells with intact membranes and damaged membranes, and propidium iodide for staining bacterial cells with damaged membranes. Cells were washed with PBS twice and resuspended in 50 µl PBS. Imaging was performed on a Nikon Eclipse TE2 inverted fluorescence microscope with a 60x (NA 1.30) oil-immersion objective (Nikon Instruments) and collected by Hamamatsu digital camera C11440 using the NIS Elements software (Nikon Instruments). When recording live video, a 35 mm uncoated dish with 14 mm glass diameter (MatTek, United States) was used. Briefly, 1 ml of exponential phase bacterial cells was collected and washed with PBS. Once Pse-MAB1 was added, the mixture was transferred onto the disk and imaged immediately on Nikon Eclipse TE2 inverted fluorescence microscope. The images were captured under bright field using a continuous imaging setting: 1 capture every 10s with a 10 min duration. The video was exported with a 50 ms interval, which means a 200× play speed.

Generation of *pseI* gene deletion mutant

Generation of the *pseI* gene deletion mutant in *A. baumannii* strain Ab8 was conducted using the CRISPR-Cas9-based genome editing platform [3]. Firstly, a 20 bp spacer ahead of the PAM sequence (5'- CGAAGCGTACGCCCAGGCTA-TGG-3') in the *pseI* gene was introduced into plasmid pSGAb-Km and confirmed by DNA sequencing. Then, 200 ng spacer-introduced pSGAb-km plasmid and 300 ng dsDNA donor repair template were electroporated into the

freshly prepared pCasAb-harboring *A. baumannii* Ab8 electrocompetent cells. After incubation on LB agar plate supplemented with 100 μg/mL apramycin and 50 μg/mL kanamycin, several colonies were randomly picked to verify the success of the genome editing by colony PCR using primers pseI-F (5'-GATCAGCGAGGCGAATTTGA-3') and pseI-R (5'-GAGCGGTGCTATTTTCAAACC-3'). Finally, both introduced plasmids in the successful *A. baumannii* mutants were cured by incubation onto an LB agar plate supplemented with 5% w/v sucrose.

Extraction of capsule

Capsule Polysaccharide (CPS) was extracted according to the method described by Tipton and Rather [4]. Bacterial strain was inoculated on LB agar plates and incubated at 37 °C for approximately 20 h. Then bacterial cells were scraped from plates with plastic loops and suspended in LB nutrient broth. The optical density of bacterial suspension was adjusted to approximately 1. Cells were harvested by centrifugation, resuspended in 200 μL lysis buffer (60 mM Tris-HCl, 50 μM $CaCl_2$, 20 mM $MgCl_2$, 3 mg lysozyme, pH 8.0) and incubated at 37 °C for 1 hr. Then 20 ng DNase and 20 ng RNase were added into the extractions, followed by an incubation at 37 °C for 0.5 hr. After another incubation with 10 μL 10% SDS at 37 °C for 0.5 hr, 150 μL lysis buffer with proteinase K were added, followed by an incubation at 37 °C for 1 hr. The extractions were centrifuged at maximum speed and the supernatant containing capsule was collected. Cold (-20 °C)75% ethanol was added into the mixture, which was then incubated at -20°C overnight to precipitate the CPS. The mixture was centrifuged at 14,000 rpm for 30 minutes at 4°C, and the supernatant was removed. The pellet was dried at 70°C, resuspended in MilliQ H_2O and stored at -20°C until use.

Mouse infection model

Survival of mice infected by *A. baumannii* strain Ab-00.191 using a mouse sepsis model was determined. In brief, *A. baumannii* strain Ab-00.191 was cultured to logarithmic phase (OD~0.6) at 37 °C in LB medium and then adjusted to the appropriate concentration in PBS. Bacterial concentrations of the inoculum were determined by plating on BHI agar plates. A 0.2 ml volume with 4 $\times 10^7$ CFU together with 100 μg of Pse-MAB1 or PBS was then injected intraperitoneally to female 6-8 weeks old inbred C57BL/6J mice (10 mice per group). Survival rate of mice was observed and recorded for 7 days post infection at 12 hours intervals.

Post-infection tissue bacterial loads were determined. Ten Mice infected as the survival experiment were anesthetized at 24 h after inoculated of bacteria. Blood and tissues including spleen, kidney, lung, liver and heart were removed aseptically. Tissues were weighed and then homogenized in sterilized PBS. Serial dilutions of tissues and blood were plated on BHI agar and incubated at 37°C for bacterial quantification. Serum levels of interleukin-1β (IL-1β), tumor necrosis factor alpha (TNF-α), and IL-6 were also determined in mice at 24 h post infection, using mouse ELISA kits (Thermo Fisher Scientific, United States).

Statistical analysis

Statistical analysis was performed using the unpaired two-sided Student's *t*-test by GraphPad Prism (San Diego, CA). Comparison of survival curves was performed by Log-rank (Mantel-Cox) test.

Supplementary Results

a)

No.1	1	2	3	4	5	6	7	8	9	10	11	12
A	0.051	0.036	0.034	0.027	0.014	0.019	0.021	0.02	0.018	0.035	0.023	0.04
B	0.026	0.024	0.012	0.022	0.02	0.022	0.01	0.024	0.029	0.024	0.036	0.036
C	0.025	0.02	0.014	0.019	0.013	0.019	0.014	0.017	0.023	0.024	0.023	0.033
D	0.024	0.015	0.025	0.021	0.024	0.021	0.023	0.019	0.018	0.017	0.019	0.028
E	0.028	0.023	0.02	0.021	0.028	0.03	0.016	0.017	0.019	0.016	0.026	0.035
F	0.024	0.021	0.025	0.021	0.025	0.017	0.021	0.027	0.02	0.032	0.023	0.033
G	0.031	0.021	0.025	0.03	0.02	0.018	0.026	0.02	0.027	0.018	0.028	0.027
H	0.03	0.049	0.032	0.034	0.036	0.025	0.061	0.047	0.04	0.035	0.042	0.762

NO.2	1	2	3	4	5	6	7	8	9	10	11	12
A	0.05	0.037	0.035	0.018	0.014	0.019	0.02	0.001	0.018	0.012	0.023	0.027
B	0.014	0.01	0.011	0.001	0.01	0.008	0.01	0.001	0.012	0.011	0.017	0.016
C	0.025	0.021	0.014	0.019	0.013	0.019	0.029	0.007	0.023	0.001	0.022	0.02
D	0.024	0.024	0.025	0.021	0.024	0.021	0.023	0.019	0.018	0.007	0.02	0.028
E	0.027	0.023	0.021	0.021	0.018	0.019	0.016	0.017	0.06	0.016	0.015	0.022
F	0.024	0.02	0.016	0.009	0.025	0.017	0.012	0.001	0.02	0.008	0.022	0.032
G	0.021	0.021	0.025	0.017	0.02	0.018	0.026	0.021	0.012	0.018	0.028	0.028
H	0.057	0.023	0.032	0.034	0.026	0.025	0.02	0.007	0.019	0.011	0.042	0.799

NO.3	1	2	3	4	5	6	7	8	9	10	11	12
A	0.031	0.037	0.024	0.018	0.014	0.019	0.03	0.001	0.018	0.011	0.037	0.027
B	0.027	0.316	0.012	0	0.009	0.009	0.01	0	0.001	0.001	0.007	0.016
C	0.039	0.021	0.033	0.018	0.012	0.019	0.015	0.002	0.01	0.001	0.012	0.02
D	0.036	0.565	0.024	0.02	0.024	0.021	0.013	0.009	0.018	0.007	0.011	0.012
E	0.027	0.024	0.021	0.021	0.018	0.019	0.016	0.017	0.01	0.015	0.015	0.013
F	0.025	0.02	0.016	0.026	0.025	0.016	0.021	0.001	0.019	0.008	0.022	0.02
G	0.031	0.021	0.026	0.017	0.019	0.018	0.027	0.011	0.012	0.018	0.016	0.018
H	0.041	0.023	0.032	0.025	0.026	0.025	0.02	0.02	0.019	0.011	0.032	0.821

NO.4	1	2	3	4	5	6	7	8	9	10	11	12
A	0.041	0.037	0.024	0.009	0.014	0.018	0.031	0.001	0.001	0.001	0.027	0.01
B	0.011	0.347	0.012	0.001	0.001	0.009	0.01	0.001	0.001	0.001	0.001	0.001
C	0.04	0.035	0.044	0.019	0.012	0.02	0.016	0.002	0.019	0.001	0.012	0.02
D	0.035	0.029	0.024	0.021	0.024	0.021	0.024	0.009	0.009	0.007	0.012	0.013
E	0.048	0.033	0.021	0.021	0.019	0.019	0.038	0.017	0.01	0.015	0.015	0.012
F	0.024	0.02	0.017	0.027	0.012	0.016	0.021	0.001	0.001	0.009	0.021	0.02
G	0.043	0.038	0.025	0.017	0.019	0.018	0.027	0.011	0.021	0.018	0.016	0.018
H	0.041	0.023	0.032	0.025	0.026	0.025	0.02	0.031	0.042	0.137	0.031	0.812

NO.5	1	2	3	4	5	6	7	8	9	10	11	12
A	0.041	0.024	0.037	0.009	0.014	0.018	0.017	0.001	0.001	0	0.012	0.01
B	0.213	0.011	0.035	0	0.001	0.009	0.01	0.001	0.001	0.001	0.001	0
C	0.018	0.016	0.669	0.019	0.012	0.019	0.016	0.001	0.019	0.002	0.012	0.02
D	0.035	0.013	0.081	0.01	0.008	0.02	0.025	0.009	0.009	0.002	0.013	0.013
E	0.019	0.017	0.021	0.021	0.018	0.032	0.019	0.001	0.01	0.015	0.015	0.012
F	0.024	0.02	0.017	0.018	0.013	0.016	0.022	0.001	0	0.008	0.021	0.02
G	0.027	0.027	0.025	0.017	0.01	0.018	0.026	0.01	0.011	0.018	0.026	0.019
H	0.02	0.033	0.032	0.025	0.025	0.026	0.02	0.015	0.011	0.033	0.032	0.713

NO.6	1	2	3	4	5	6	7	8	9	10	11	12
A	0.027	0.024	0.017	0.009	0.024	0.017	0.001	0.001	0.001	0.001	0.012	0.01
B	0.011	0.283	0.002	0.001	0.001	0.008	0.001	0	0.001	0.001	0.001	0.001
C	0.018	0.015	0.028	0.019	0.011	0.019	0.001	0.001	0.001	0.001	0.012	0.02
D	0.035	0.013	0.018	0.01	0.009	0.02	0.001	0.009	0.001	0.007	0.012	0.013
E	0.028	0.017	0.02	0.021	0.018	0.013	0.001	0.001	0.001	0.015	0.014	0.012
F	0.024	0.02	0.017	0.027	0.013	0.016	0.001	0.001	0.001	0.009	0.021	0.02
G	0.027	0.027	0.025	0.017	0.01	0.019	0.001	0.01	0.001	0.018	0.015	0.019
H	0.036	0.033	0.022	0.026	0.025	0.025	0.001	0.015	0.001	0.022	0.032	0.762

NO.7	1	2	3	4	5	6	7	8	9	10	11	12
A	0.043	0.024	0.029	0.009	0.024	0.03	0.042	0.007	0	0.001	0.049	0.01
B	0.011	0.015	0.002	0.001	0.001	0.008	0	0.001	0.001	0.001	0.001	0.022
C	0.018	0.015	0.018	0.018	0.011	0.019	0.478	0.007	0.001	0.007	0.012	0.02
D	0.035	0.026	0.018	0.019	0.008	0.02	0.54	0.009	0.001	0.008	0.012	0.013
E	0.028	0.016	0.021	0.021	0.018	0.012	0.018	0.001	0.52	0.001	0.014	0.012
F	0.024	0.02	0.016	0.026	0.013	0.016	0.001	0.001	0.015	0.008	0.021	0.02
G	0.027	0.028	0.025	0.028	0.021	0.045	0.018	0.01	0.001	0.017	0.015	0.019
H	0.036	0.023	0.022	0.025	0.024	0.493	0.035	0.015	0.024	0.01	0.021	0.725

NO.8	1	2	3	4	5	6	7	8	9	10	11	12
A	0.031	0.023	0.029	0.022	0.025	0.03	0.016	0.002	0.017	0	0.015	0.019
B	0.025	0.015	0.001	0.001	0.001	0.008	0.015	0	0.001	0	0.001	0.013
C	0.032	0.036	0.017	0.018	0.011	0.018	0.016	0.001	0.017	0.007	0.012	0.02
D	0.034	0.026	0.017	0.019	0.009	0.019	0.026	0.008	0.001	0.008	0.013	0.012
E	0.028	0.017	0.021	0.02	0.017	0.012	0.018	0.001	0.014	0	0.014	0.012
F	0.023	0.02	0.027	0.052	0.013	0.016	0.018	0.064	0.014	0.008	0.02	0.021
G	0.027	0.027	0.025	0.028	0.021	0.019	0.017	0.01	0.001	0.016	0.015	0.019
H	0.036	0.033	0.022	0.026	0.024	0.017	0.018	0.014	0.025	0.02	0.021	0.704

NO.9	1	2	3	4	5	6	7	8	9	10	11	12
A	0.031	0.023	0.017	0.011	0.012	0.014	0.016	0.001	0.017	0.001	0.015	0.007
B	0.015	0.015	0.007	0	0.001	0.008	0.015	0.001	0.017	0.001	0.001	0.013
C	0.02	0.035	0.018	0.018	0.011	0.018	0.016	0.001	0.016	0.008	0.012	0.001
D	0.034	0.026	0.017	0.019	0.018	0.019	0.026	0.447	0.018	0.008	0.013	0.012
E	0.028	0.017	0.021	0.02	0.017	0.03	0.018	0.021	0.015	0.018	0.014	0.012
F	0.023	0.021	0.026	0.018	0.013	0.016	0.028	0.028	0.024	0.009	0.042	0.021
G	0.028	0.027	0.025	0.028	0.021	0.02	0.018	0.024	0.021	0.016	0.026	0.019
H	0.025	0.02	0.021	0.026	0.024	0.018	0.018	0.015	0.024	0.019	0.021	0.874

NO.10	1	2	3	4	5	6	7	8	9	10	11	12
A	0.031	0.033	0.017	0.01	0.011	0.014	0.015	0.001	0.001	0	0.015	0.007
B	0.024	0.001	0.007	0.001	0.001	0.001	0.001	0.001	0.001	0.001	0.001	0.001
C	0.035	0.053	0.017	0.018	0.011	0.018	0.017	0.001	0	0.007	0.011	0.016
D	0.034	0.026	0.016	0.01	0.018	0.019	0.026	0.001	0.001	0.008	0.014	0.013
E	0.027	0.017	0.022	0.02	0.017	0.012	0.019	0.001	0.015	0.001	0.014	0.012
F	0.034	0.021	0.011	0.017	0.013	0.001	0.008	0.001	0.001	0.009	0.008	0.021
G	0.027	0.028	0.025	0.029	0.021	0.02	0.018	0.001	0.008	0.016	0.026	0.019
H	0.025	0.021	0.021	0.011	0.009	0.018	0.009	0.001	0.001	0.008	0.021	0.775

b)

No.	OD450nm	Pse-MAB
1	0.069	
2	0.566	Pse-MAB1
3	0.032	
4	0.043	
5	1.052	Pse-MAB2
6	0.033	
7	0.42	Pse-MAB3
8	0.865	Pse-MAB4
9	0.692	Pse-MAB5
10	0.713	Pse-MAB6
11	0.768	Pse-MAB7
Negative	0.071	
PBS	0.053	
Positive	1.23	

Figure S1. Screening of hybridoma cell lines producing Pse-specific antibodies. a) First-round screening of the Pse-specific antibodies-producing hybridoma cell lines. The Pse-specific antibodies in the cell supernatants of the 93*10 monoclonal cells tested by ELISA using Pse-BSA conjugates as captures, and each number indicated one cell line. The purple indicated negative control, the blue indicated PBS and the red indicated positive control. A total of 11 hybridoma cell lines (green) were screened as Pse-specific antibodies positive cell lines. b) Second-round screening of the Pse-specific antibodies-producing hybridoma cell lines. The Pse-specific antibodies in the cell supernatants of the 11 cell lines were further tested by ELISA using Pse-BSA conjugates as captures. A total of 7 hybridoma cell lines were identified as Pse-specific antibodies-producing hybridoma cell lines.

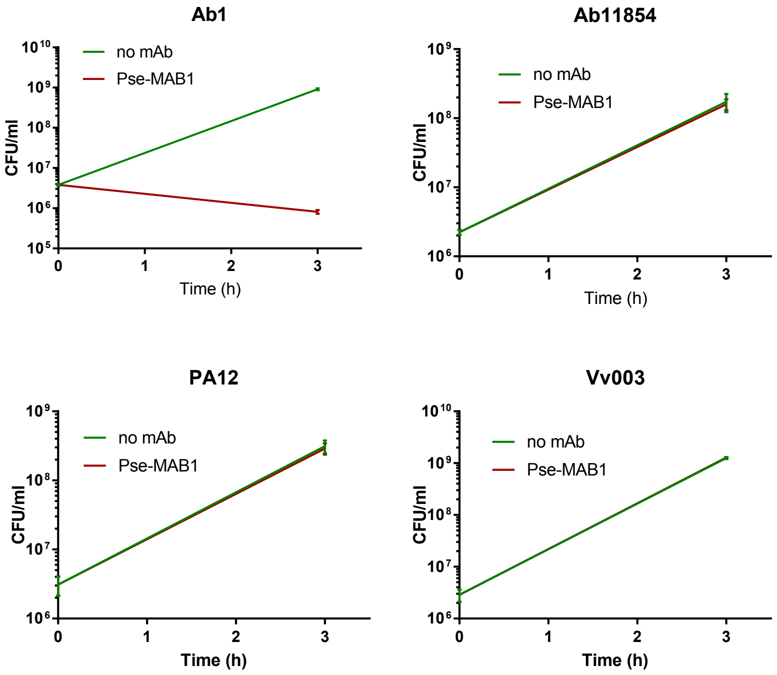

Figure S2. Growth curve of *A. baumannii* strain Ab1, Ab11854, *P. aeruginosa* strain PA12 and *V. vulnificus* strain Vv003 in the absence and presence of Pse-MAB1 (1 nM). CFUs were measured at 3 h after the addition of Pse-MAB1.

Uncategorized References

1. Andolina, G., et al., *Metabolic Labeling of Pseudaminic Acid-Containing Glycans on Bacterial Surfaces.* ACS Chemical Biology, 2018. **13**(10): p. 3030-3037.
2. Wei, R., et al., *Synthetic Pseudaminic-Acid-Based Antibacterial Vaccine Confers Effective Protection against Acinetobacter baumannii Infection.* ACS Cent Sci, 2021. **7**(9): p. 1535-1542.
3. Wang, Y., et al., *A Highly Efficient CRISPR-Cas9-Based Genome Engineering Platform in Acinetobacter baumannii to Understand the H2O2-Sensing Mechanism of OxyR.* Cell Chemical Biology, 2019. **26**(12): p. 1732-1742.e5.
4. Tipton, K.A. and P.N. Rather, *Extraction and Visualization of Capsular Polysaccharide from Acinetobacter baumannii*, in *Acinetobacter baumannii: Methods and Protocols*, I. Biswas and P.N. Rather, Editors. 2019, Springer New York: New York, NY. p. 227-231.
